# Gender Concordance and Patient Outcomes in Indian Telemedicine: Retrospective Cross-Sectional Quantitative Study of 286,000 Consultations

**DOI:** 10.2196/78311

**Published:** 2026-01-20

**Authors:** Nafisa Vaz, Vishalkumar Jani

**Affiliations:** 1 Healthcare Management Department, Goa Institute of Management, Sattari, Sanquelim, Goa, 403505, India, 91 8888885033; 2 Practo Technologies Pvt Ltd., Bangalore, Karnataka, India

**Keywords:** gender concordance, telemedicine, India, patient satisfaction, clinical outcomes, culturally sensitive specialties, physician-patient dyads

## Abstract

**Background:**

Gender concordance (GC) between patients and physicians has been linked to trust and satisfaction in traditional health care. However, its role in telemedicine, especially in culturally complex settings like India, is underexplored. In India’s culturally diverse and gender-sensitive context, understanding GC becomes particularly relevant for specialties such as gynecology, dermatology, psychiatry, and urology, where discussions often involve intimate or stigmatized concerns. Despite rapid telemedicine expansion, little empirical evidence exists on whether GC affects patient-reported outcomes in this context.

**Objective:**

This study examined whether GC significantly influences patient satisfaction and self-reported recovery in teleconsultations across India, with a focus on specialty-specific effects in culturally sensitive specialties.

**Methods:**

We conducted a retrospective cross-sectional analysis of 286,196 anonymized teleconsultation records from a national telemedicine platform (January 2023–December 2024) spanning across 20 medical specialties using binary logistic regression. Records missing gender or satisfaction data were excluded from the analysis; recovery analyses included only consultations with completed day-21 follow-up surveys (n=1170, 0.4%). Outcomes included patient satisfaction (ratings 4‐5 on a five-point scale) and self-reported recovery at follow-up. Logistic regression models (Stata 17.0) tested associations between GC and outcomes, controlling for consultation time, duration, and physician experience. Subgroup analyses were conducted for the top 5 specialties. Each record included key data on consultation duration, timing, physician experience, specialty type, patient satisfaction rating, and self-reported recovery status. The study excluded the pediatrics specialty from the analysis to control for the parental bias.

**Results:**

Of the 286,196 consultations, 164,008 (60.4%) were gender-concordant. Overall, 261,213 of 286,196 (91.3%) patients reported good satisfaction. GC had a statistically significant negative association with patient satisfaction (odds ratio [OR] 0.87, 95% CI 0.85‐0.90; *P*<.001). Across gender, the male doctor received higher satisfaction. In gynecology, female patient–female doctor pairs had significantly higher odds of reporting recovery (OR 4.53, 95% CI 0.8‐25.3; *P*=.099). Overall, consultation timing (OR 0.99, 95% CI 0.998‐0.999; *P*<.001) and patient satisfaction (OR 20.13, 95% CI 12.06‐35.38; *P*<.001) were stronger predictors of self-reported recovery than GC.

**Conclusions:**

GC in telemedicine has a context-dependent impact. While it does not independently predict clinical recovery, it meaningfully shapes patient satisfaction. These findings highlight that gender sensitivity training and context-specific communication approaches may enhance telemedicine experiences in culturally sensitive domains. Integrating awareness of gender dynamics into telehealth design and policy could strengthen patient trust and engagement in virtual care. Future research should explore specialty-specific dynamics and improve follow-up response rates to better assess clinical outcomes.

## Introduction

### Telemedicine and Gender Dynamics in the Indian Context

The COVID-19 crisis has triggered a revolutionary change in health care delivery and expedited the worldwide penetration of telemedicine as an acceptable mode of consultation [1]. In India, with sharp health care access inequalities, especially across rural and socioeconomically disadvantaged populations, telemedicine presented itself as a vital mechanism for overcoming geographic and resource-based barriers [2,3]. Latest figures indicate a sweeping rise in virtual consults, with millions of patients depending on telehealth facilities for medical advice, diagnosis, and treatment [4].

Despite the operational success and scalability of telemedicine in India, patient experience and perceived quality are varying [5]. While aspects like connectivity, clinical competence, and usability of the platform are extensively researched [2,6], one aspect that is not well examined is the influence of gender in the construction of the virtual doctor-patient interaction. More specifically, the way the gender match or mismatch between patient and provider, also known as gender concordance (GC), has an effect in the Indian telemedicine context.

GC, alignment between the gender identity of the health care provider and the patient, has been linked to improved trust, communication, and outcomes, particularly in intimate specialties like gynecology and psychiatry [7]. But the impact of GC in telemedicine, a medium that lacks presence and is based primarily on audio-only interactions, is not well understood, particularly against the Indian backdrop [8].

India’s sociocultural landscape presents an additional level of complexity. Strongly rooted gender norms of modesty and patriarchal formations tend to shape health care–seeking behavior [7-10]. Women will be reluctant to share certain symptoms with a male physician, and male patients will find female health care providers more empathic across certain specialties [11,12]. Such factors pose essential questions regarding the impact of GC on patient satisfaction and perceived effectiveness in telemedicine.

Recent telehealth studies [7,11,13] show that GC may influence trust and comfort differently across cultural contexts, but evidence from non-Western settings remains scarce. Few large-scale analyses have examined patient-reported outcomes in virtual care, especially in India. Previous studies in this area have been carried out in Western societies with varying cultural values and health care infrastructure [14,15]. Consequently, it is not possible to transfer findings from such studies directly to India. Moreover, studies so far have concentrated mostly on clinical results or communication patterns, with a lack of proper incorporation of patient-reported measures like satisfaction or self-perceived recovery. There is an utmost need for large-scale evidence from a contextualized setting that can be used for developing culture-sensitive design and implementation of telemedicine services for India.

This paper attempts to fill this lacuna by investigating whether GC is a predictor of 2-principal patient-reported outcomes, consultation satisfaction, and self-reported recovery, in telemedicine consultations across a broad spectrum of medical specialties in India. Using a database of more than 286,000 anonymous telemedicine consultations conducted by one of India’s biggest telemedicine platforms, we explore the interaction between gender dynamics and other factors, including consultation timing, physician experience, and specialty type, in shaping patient perceptions of quality of care.

### GC in Health Care: Global Evidence

GC, the match between a patient’s and a provider’s gender, has typically been linked with increased patient trust, satisfaction, and communication in conventional health care situations, physical face-to-face consultations, especially in developed economies [6,15,16]. The important point to note here is that literature about GC in clinical settings has been using self-reported sex or gender by the patients and doctors as a gender variable and does not really differentiate between gender and sex of a person like some other social science studies would have done. For instance, GC in primary care is related to longer consultation times and more patient-oriented communication [16], and, in oncology, female patients tend to be more comfortable with female providers because of impressions of empathy and emotional support [17].

Yet the results are still unclear. Previous studies [14,18] discovered minimal or no effect of GC on clinical outcomes or satisfaction, which suggests that GC by itself is not a strong predictor of health outcomes. One of the studies also stresses the differences in GC effects depending on the clinical specialty and setting in a related systematic review [15].

With the development of telemedicine, questions arise as to whether the impact of GC is present in the virtual setting. While nonverbal cues are diminished in teleconsultations, there is evidence that rapport and trust can still be established [19]. When voice-only consultation is used, as it is in resource-poor environments such as rural India, GC can facilitate comfort and disclosure by alleviating gender-based communication barriers [18]. However, the inconclusive global evidence underscores the necessity for context-oriented, culture-sensitive studies.

### Sociocultural Context and Gender Preferences in India

India is a specific context to study GC because of the entrenched gender norms, especially in specialties with sensitive health matters [20]. Research reveals that female patients are likely to seek female providers, particularly in psychiatry and gynecology, where personal revelation is necessary [2,14]. Hofstede cultural dimensions, specifically masculinity-femininity and power distance, can explain why the health care expectations of the patients in India tend to be influenced by gendered constructs of authority and nurturing [21]. These sociocultural undercurrents can shape whether GC is likely to build trust or create new tensions.

### Specialty-Specific Insights

GC’s impact varies by specialty, widely preferred in gynecology for perceived empathy, considered in pediatrics, and supportive of disclosure in mental health, especially for women [6,8,21,22]. Those differences highlight the need to examine GC as a specialty-specific rather than a general concept. For pediatric consultations, GC was determined based on the gender of the primary caregiver who initiated the consultation, minimizing potential bias from child accounts.

### Empirical Gaps in Indian Telemedicine

Despite growing health care reach and diminished geographic barriers [1,3], telemedicine’s capacity for supporting patient-provider relationships within gender-sensitive frameworks is uncertain. More satisfaction was evidenced in GC-based video interactions by Verma et al [23], but outcomes of recovery did not vary significantly. More critical factors, for example, consultation duration and doctor experience, could play an even more powerful role in shaping outcomes, and so analyses including these factors with GC as control variables are needed.

### Empirical Lacunae in Indian Research

With the increasing utilization of telemedicine in India [5], there is still a scarcity of empirical evidence regarding the impact of GC on the delivery of health care virtually, especially from the patient’s side. Female patients in rural and traditional areas can avoid or underdeclare their problems during interactions with male practitioners, particularly for reproductive or sexual health [9]. Previous studies [2,3,22,24] underscored the importance of system design with a gender awareness that ensures equal access. No large-scale, quantitative studies so far have thoroughly analyzed the effect of GC on both satisfaction and health outcomes in telemedicine in India.

With the mixed international evidence, this research will bridge the gaps in existing research by investigating the outcomes of GC for patient satisfaction and self-perceived recovery from teleconsultation in various medical specialties in India. The issue under investigation is relevant to both policy and practice of allocation algorithms, provider communication and behavior, and training needs for providers working for telemedicine platforms in India. The research is relevant to all telemedicine platforms—public and private.

### Study Aims

This research is directed to assess the role of GC between patient and doctor in customer satisfaction (CSAT) and patient-reported medical efficacy (PRME), recovery rate, in the Indian telemedicine context. The study hopes to provide evidence-based insights into the telemedicine encounter as influenced by sociodemographic congruence between providers and patients. We expect the findings to have direct practical applications for telehealth platform design, provider training, and patient matchmaking algorithms. Guided by expectation confirmation theory (ECT) and the patient-centered care model (PCCM), this study examines whether GC predicts (1) patient satisfaction and (2) self-reported recovery in teleconsultations across India. We hypothesize that GC may have specialty-specific effects, particularly in gynecology.

## Methods

### Data Source and Study Design

This study draws on a dataset provided by one of the leading private Indian telemedicine platforms, recognized for its extensive reach and service diversity across specialties [25]. The platform was selected due to its nationwide footprint and volume of consultations, making it a strong representative sample for analyzing virtual care patterns across India. As of 2024, the platform reports over 400 million registered users across 22 states, positioning it among the largest telehealth providers in the country. The platform provides close to 2 million teleconsultations annually.

This retrospective cross-sectional study used secondary, anonymized data from a leading Indian telemedicine platform. The dataset covered consultations from January 2023 to December 2024.

### Sample Selection and Data Processing

The dataset consists of 286,196 telemedicine consultations from various specialties and systems of medicine, such as Allopathic, Ayurveda, and Homeopathy. The current study used the anonymized data provided by the telemedicine platform. The organization has robust processes to anonymize the data and does not share any personally identifiable information, even within the organization. The data were transferred to authors in a spreadsheet where each transaction was identified with a unique identification number. For all purposes, the data were secondary in nature for the investigators.

The platform, over the period of 2 years, had done close to 3 million teleconsultations. However, for the study purposes, the platform shared only the data for consultations for which the patient had responded to the patient satisfaction survey (CSAT). The health outcome medical efficacy (PRME) survey started in April 2024, and hence it is available in very limited proportion. The inclusion of transactions was based on the fact that the CSAT or PRME survey was responded back by the patient because outcome data were important for the study. To utilize the provided data, authors had to filter the data. The transactions with reported CSAT and PRME were separated. Patients’ and doctors’ genders were reported, so GC was derived as a variable and utilized to categorize the transactions.

### Dataset Screening Process

A structured screening process was applied to 286,196 for missing data and to form the analytical sample.

- Exclusion criteria for satisfaction analysis were as follows:
  - Missing patient gender (n=14,578)
  - Missing doctor gender (n=151)
  - Unclear satisfaction rating response (n=3)
- Exclusion criteria for recovery analysis were as follows:
  - All consultations without a completed day-21 recovery follow-up (n=284,874)
  - Pediatric consultations (n=29,288) due to caregiver-provided responses

Patients accessed the platform through a web or mobile interface. The current structure of the telemedicine platform automatically assigns a doctor to a patient based on the specialty or symptom that the patient enters into the system. An algorithm that is based on the availability of doctors on the platform at that time connects a patient with a doctor. So, there is no choice from either the doctor or patient side involved in matching.

The current study excludes the pediatrics consultations from the assessment of the impact of GC on the CSAT and PRME. The reason here is the response gets confounded by the parent or parents’ preferences or biases. The patient himself or herself is not the one responding to either of the surveys.

### Variables and Measures

#### Dependent Variables

##### Patient Satisfaction [csat_good]

This binary variable indicates whether the patient reported high satisfaction with the consultation. This variable is created from the 1-5 scale-based question asked at the end of each consultation. The ratings of 1, 2, and 3 were considered to be dissatisfied (csat_good=0), and the ratings of 4 and 5 were considered to be satisfied (csat_good=1).

##### PRME-Recovery Rate

This binary variable indicates whether the patient reported improvement in symptoms after consultation. This recovery rate question is floated to all patients on the 21st day after consultation. Patients who reported recovery are captured as prme_good=1, and 0 otherwise.

### Independent Variables

#### GC [gender_cc]

This binary variable indicates whether the patient’s gender matched the physician’s gender. The platform collects this demographic information as part of preconsultation user inputs; patients self-report their sex when registering or booking a consultation. Physicians do not collect or verify this information during the consultation process. While the system technically captures biological sex, we acknowledge that much of the existing literature in this domain uses the term “GC” rather than “sex concordance.” In line with prevailing academic convention, we continue using the term GC throughout this study, while recognizing this conceptual limitation in our definition of the research question.

#### Time of Day [time_day]

This captures consultation timing, categorized into morning and night. The platform uses a 9 AM to 9 PM window as usual day timing from the doctor’s practice perspective. Hence, consultation happening in this time period is marked as time_day=1.

#### Consultation Duration [total_dura]

This was the total duration [in seconds] of the teleconsultation.

#### Physician Experience [dr_experience]

This was measured in years of practice.

### Data Analysis

The study started analysis with the finding of proportions of transactions reported with positive CSAT and positive PRME across patient-doctor gender-concordant dyads and nonconcordant dyads. The study employed a *z* test to check if the 2 groups were statistically different in CSAT or PRME.

To assess the impact of GC on patient outcomes, the study employed a logistic regression model. Exponentiated coefficients (odds ratios [OR]) were reported to interpret the impact of GC and other control variables. The analysis also included the regressions at the specialty level. These specialties were selected by consultation volume. These were identified using the metric of total number of completed consultations within the study period. Logistic regression analysis requires an adequate number of outcome events relative to the number of predictor variables to ensure the stability and reliability of parameter estimates. Following conventional guidelines, a minimum of 10 outcome events per predictor variable is recommended to avoid overfitting and ensure model validity [24]. Additionally, the Central Limit Theorem supports a minimum sample size of 30 as a general rule of thumb for approximating normality in sampling distributions [26]. These principles jointly informed the decision to set 30 observations as the minimum threshold for conducting specialty-specific regression analyses in this study. This gave us 9 specialties for CSAT and 7 specialties for PRME. However, the result tables reported only the 5 specialties that had the highest number of responses available for CSAT or PRME. Analyses were conducted using Stata (version 17.0; StataCorp LLC). Binary logistic regression models assessed associations between GC and 2 outcomes, patient satisfaction, and self-reported recovery, while controlling for consultation duration, physician experience, and time of day. Specialty-level regressions were conducted where event counts were ≥30.

### Ethical Considerations

This study was approved by the Board of Research Ethics (BORE) of Goa Institute of Management (Approval No. GIM/BHCM042507). The study was conducted in compliance with ethical research standards. Since the data were anonymized and obtained with necessary permissions from the telemedicine platform, no personally identifiable information was accessed. The research adheres to data protection laws and ethical guidelines related to patient confidentiality. This retrospective study was approved by the institutional review board of Goa Institute of Management. Data used were deidentified secondary records collected with informed consent for service use; the platform’s terms permit anonymized secondary analyses. All identifiers were removed prior to researcher access; only aggregate data were analyzed. No compensation was involved. No identifiable images or patient details are included.

## Results

### Overview of the Sample

Of the 286,196 consultations analyzed, 60.4% (n=164,008) were gender concordant and 47.8% (n=129,848) involved female patients. Table 1 shows the specialty-wise shares in total transactions. Pediatrics is shown in descriptive statistics but excluded from outcome analyses due to caregiver response bias.

**Table 1.** Summary of data and variables.

Number of teleconsultations	Count (N=286,196)
Patient age (y), median (IQR)	30.0 (25-37)
Patient sex, n (%)	
Male	141,670 (52)
Female	129,848 (48)
Doctor experience (y), median (IQR)	8.0 (5-12)
Doctor sex, n (%)	
Male	169,363 (59)
Female	116,682 (41)
Specialty, n (%)	
Ayurveda	2021 (0.7)
Cancer	600 (0.2)
Cardiology	5290 (1.8)
Dental	3329 (1.2)
Dermatology	38,824 (14)
Diabetology	3729 (1.3)
Diet and nutrition	6069 (2.1)
Ear, Nose, Throat	11,282 (3.9)
Eye and Vision	5506 (1.9)
Gastroenterology	5654 (2)
General physician	65,794 (23)
General surgery	967 (0.3)
Gynecology	37,876 (13)
Homeopathy	2754 (1)
Nephrology	1643 (0.6)
Neurology	7212 (2.5)
Orthopedics	11,871 (4.1)
Pediatrics	29,288 (10)
Physiotherapy	1427 (0.5)
Psychiatry	5688 (2)
Psychological counseling	3993 (1.4)
Pulmonology	3216 (1.1)
Rheumatology	777 (0.3)
Sexology	10,192 (3.6)
Stomach and digestion	10,176 (3.6)
Urology	9798 (3.4)
Unknown	1220 (0.4)
Time of consultation, n (%)	
Peak hours—day (9 AM to 9 PM)	193,612 (68)
Nonpeak hours—night (9 PM to 9 AM)	92,583 (32)
Consultation duration (s), median (IQR)	382 (237-600)
CSAT^a^ score (1-5), n (%)	
1	12,461 (4.4)
2	3732 (1.3)
3	8787 (3.1)
4	39,024 (14)
5	222,191 (78)
CSAT (good vs bad), n (%)	
Bad	24,980 (8.7)
Good	261,213 (91)
PRME^b^ responses, n (%)	1322 (0.46)
PRME (good vs bad), n (%)	
Not recovered	322 (24)
Recovered	1000 (76)
GC^c^, n (%)	
Discordant	107,369 (40)
Concordant	164,008 (60)

a CSAT: customer satisfaction.

b PRME: patient-reported medical efficacy.

c GC: gender concordance.

Table 2 shows that 135,755 out of 148,803 (91.23%) transactions with a gender-concordant patient-doctor dyad reported good CSAT compared with 86,680 out of 93,971 (92.24%) transactions where doctor-patient GC was not present. Even when the sample was categorized for patient gender, it showed similar results. Gender-concordant transactions showed lower CSAT compared with nonconcordant transactions.

**Table 2.** Statistical difference in CSAT^a^ and PRME^b^ across different groups.

CSAT			PRME		
Group	GC^c^=0, n/N (%)	GC=1, n/N (%)	*z* score (*P* value)	GC=0, n/N (%)	GC=1, n/N (%)	*z* score (*P* value)
All	86,680/93,971 (92.24)	135,755/148,803 (91.23)	8.753 (<.001)	389/502 (77.49)	495/669 (73.99)	1.38 (.17)
Female patients	51,511/55,808 (92.3)	55,057/61,003 (90.25)	12.358 (<.001)	233/304 (76.64)	203/261 (77.78)	–0.32 (.75)
Male patients	35,169/38,163 (92.19)	80,698/87,800 (91.87)	1.469 (.14)	156/198 (78.79)	292/408 (71.57)	1.9 (.06)

a CSAT: customer satisfaction.

b PRME: patient-reported medical efficacy.

c GC: gender concordance.

For PRME, the available transactions were only 1322, and after excluding the pediatrics specialty, the available transactions were 1199. Out of the available data on PRME, 923 of 1199 (77.01%) transactions have good PRME; that is, 77.01% patients reported that they had recovered. Out of these 1199 transactions, 1170 transactions had data on patient and doctor gender. Among these, 669 (57.17%) transactions had patient-doctor GC. Table 2 shows that gender-concordant cohort of transactions reported that PRME is 495 out of 669 (73.99%), compared with 388 out of 501 (77.49%) in the nonconcordant cohort. This difference is not statistically significant, unlike what we found with CSAT. Patient gender-wise categorization also found insignificant difference between concordant and nonconcordant cohorts. However, for the female patient cohort, the gender-concordant group reported higher PRME compared to the nonconcordant group. In contrast, it was the other way around for the male patient cohort.

Table 3 shows that, on average, male patients report higher satisfaction compared with their female counterparts, and male doctors receive better satisfaction scores compared with female doctors. However, irrespective of patient gender, a gender-discordant dyad results in a better CSAT score.

**Table 3.** CSAT^a^ for different doctor genders as per patient genders.

	Patient gender		
Doctor gender	All patients, n/N (%)	Female patients, n/N (%)	Male patients, n/N (%)
All doctors	222,564/242,914 (91.6)	106,681/116,935 (91.23)	115,883/125,979 (91.98)
Female doctors	12,398/13,713 (90.41)	7678/8592 (89.36)	4720/5121 (92.17)
Male doctors	210,166/229,201 (91.69)	99,003/108,343 (91.38)	111,163/120,858 (91.98)

a CSAT: customer satisfaction.

### GC and Patient Satisfaction

This section is focused on the logistic regression results assessing the impact of GC and other independent variables on the CSAT. The study employed logistic regression on the overall sample and separately for 9 specialties individually. Table 4 reports the regression results for the overall sample and 5 specialties that had the highest number of transactions with CSAT reported.

**Table 4.** Relationship between CSAT^a^ and gender concordance in telemedicine.

csat_good					
Variable	All, OR^b^ (95% CI; *P* value)	General physician, OR (95% CI; *P* value)	Gynecology, OR (95% CI; *P* value)	Dermatology, OR (95% CI; *P* value)	Orthopedics, OR (95% CI; *P* value)	ENT, OR (95% CI; *P* value)
Gender concordance	0.874 (0.81-0.94; <.001)	0.9796 (0.92-1.04; .49)	1.079 (0.97-1.20; .16)	0.917 (0.85-0.98; .02)	1.023 (0.89-1.18; .74)	0.841 (0.73-0.97; .02)
Consultation during office hours	1.081 (1.04-1.12; <.001)	1.114 (1.05-1.18; <.001)	1.038 (0.97-1.11; .31)	1.052 (0.98-1.13; .19)	0.867 (0.74-1.02; .08)	1.229 (1.06-1.42; <.001)
Duration of the consultation	1.0004 (1.0003-1.0005; <.001)	1.0003 (1.0002-1.0004; <.001)	1.001 (1.001-1.001; <.001)	1.001 (1.001-1.001; <.001)	1.000 (1.00-1.00; .05)	1.000 (1.00-1.00; .09)
Doctor experience	1.002 (1.00-1.002; .11)	1.003 (1.00-1.01; .06)	1.001 (1.00-1.01; .63)	0.995 (0.99-1; .16)	0.955 (0.93-1; <.001)	0.985 (0.97-1; .008)
Intercept	9.46 (8.55-10.47; <.001)	10.235 (8.96-11.69; <.001)	5.5912 (4.74-6.6; <.001)	8.6154 (7.28-10.2; <.001)	16.6125 (13.43-20.55; <.001)	11.9619 (10.27-13.93; <.001)
N	228,640	65,174	36,185	38,183	11,770	11,213

a CSAT: customer satisfaction.

b OR: odds ratio.

In the full sample, gender discordance was a statistically significant predictor of patient satisfaction (OR 0.874, 95% CI 0.85‐0.90; *P*<.001), suggesting that the presence of GC results in a lower satisfaction rate. This aligns with the findings of Table 2, showing that female patient-male doctor and male patient-female doctor dyads showed higher satisfaction rates.

At the specialty level, GC was associated with lower satisfaction in dermatology (OR 0.917, 95% CI 0.85-0.98; *P*=.02) and ENT (OR 0.841, 95% CI 0.73-0.97; *P*=.02). In the specialty of Gynecology, where patient sensitivity about doctor gender is high [27,28], GC showed a positive but statistically nonsignificant association with satisfaction. The effect of GC was not uniform, suggesting that specialty context moderates its influence on patient experience.

Except for orthopedics, all other specialties showed that transaction happening during 9 AM to 9 PM had a positive impact on the CSAT. Except for the general physician group, other specialties showed that the younger (less experienced) the doctor, the better the CSAT rate.

### GC and PRME

Table 5 reports the logistic regression results for the impact of GC on the PRME—the recovery rate. It presents 7 regressions: the first 2 for the whole sample for which all required variables were available, and the next 5 were separate specialty levels. The first model included CSAT as one of the explanatory variables, whereas the other 6 models did not include it.

**Table 5.** Relationship between patient-reported medical efficacy and gender concordance in telemedicine.

	prme_good						
Variable	All, OR^a^ (95% CI; *P* value)	All, OR (95% CI; *P* value)	General physician, OR (95% CI; *P* value)	Gynecology, OR (95% CI; *P* value)	Dermatology, OR (95% CI; *P* value)	Orthopedics, OR (95% CI; *P* value)	ENT, OR (95% CI; *P* value)
Customer satisfaction	20.1334 (8.11-50.02; <.001)	—^b^	—	—	—	—	—
Gender concordance	0.8492 (0.63-1.15; .29)	0.8401 (0.64-1.01; .21)	0.855 (0.51-1.43; .55)	4.53 (0.75-27.26; .099)	1.102 (0.6-2.03; .76)	1.168 (0.33-4.11; .81)	1.733 (0.62-4.83; .29)
Consultation during office hours	1.142 (0.82-1.59; .43)	1.2524 (0.93-1.69; .14)	1.492 (0.84-2.64; .17)	2.099 (0.77-5.75; .15)	1.324 (0.68-2.57; .41)	0.423 (0.09-2.09; .29)	0.716 (0.23-2.28; .57)
Duration of the consultation	0.999 (0.99-1.00; <.001)	0.999 (0.99-1.00; .001)	0.999 (0.99-1.00; .18)	1.001 (1.00-1.002; .18)	1 (0.99-1.002; .95)	0.998 (0.995-1; .17)	0.998 (0.99-1; .19)
Doctor experience	1.0032 (0.98-1.02; .75)	1.007 (0.99-1.03; .44)	0.998 (0.97-1.02; .87)	1.09 (1.00-1.19; .04)	0.965 (0.91-1.02; .20)	0.999 (0.81-1.19; .99)	1.054 (0.97-1.14; .19)
Intercept	0.2854 (0.2-0.42; <.001)	3.5164 (2.4-5.15; <.001)	5.2211 (3.16-8.62; <.001)	0.1725 (0.02-1.58; .12)	3.448 (1.47-8.1; .005)	6.9175 (0.00-9.98; .91)	2.7454 (0.68-11.06; .16)
N	1170	1170	430	114	243	46	79

a OR: odds ratio.

b not applicable.

Based on the 1170 transactions, GC had no statistically significant effect on overall recovery outcomes (OR 0.84, 95% CI 0.64‐1.1; *P*=.21). While marginal significance was noted in gynecology (*P*=.099), given the small number of transactions (n=1170) for recovery follow-ups, findings must be interpreted cautiously.

The first model showed that patient satisfaction was the strongest and most consistent predictor of self-reported recovery (OR 20.13, 95% CI 12.06‐35.38; *P*<.001).

Longer consultations were weakly correlated with satisfaction, not recovery. The experience of a doctor did not show a positive impact on the patient-reported recovery rate, except in the gynecology specialty.

## Discussion

### Principal Findings

This study examined the impact of GC on patient satisfaction and self-reported recovery in more than 286,000 Indian telemedicine consultations. Contrary to conventional assumptions [7], GC did not uniformly enhance satisfaction or recovery. In fact, nonconcordant dyads reported higher satisfaction, challenging existing evidence and expectations of demographic matching improving health care experiences [6,15,16].

These findings align with 2 theories: ECT and PCCM.

ECT explains how satisfaction arises when experiences exceed expectations [26,27], as seen in male patient-female doctor dyads. In concordant dyads, higher expectations may have gone unmet, reducing perceived trust [29]. Multiple studies indicate that male patients interacting with female physicians frequently experience their expectations being exceeded, particularly in the domains of interpersonal communication and empathy [30], leading to higher trust. Research reveals that male patient-female doctor dyads are often characterized by higher patient-reported satisfaction and quality scores, largely due to the “expectation-surprise” effect: male patients, who may hold lower initial expectations of female physicians’ technical and interpersonal abilities, report greatly enhanced perceptions when those expectations are surpassed in real clinical interactions [13]. Female doctors are consistently highlighted for their superior listening, attentiveness, and clarity, which not only challenge prevailing gender stereotypes but also foster increased trust and comfort among male patients. This dynamic has been observed across different care settings and cultural contexts, underscoring the potential benefits of such cross-gender provider-patient pairings for improving patient satisfaction and health care quality [31]. These superseded expectations may meet with better treatment adherence, leading to significantly different recovery rates in male patient-female doctor dyads compared to male-male dyads.

The female patient, and the overall sample, showed higher satisfaction rates when consulted with a male doctor, which is in corroboration with general perception of men being preferred for technical roles [32] and previous evidence of this female patients rating male doctors better than the female doctors [33].

**Figure 1.**
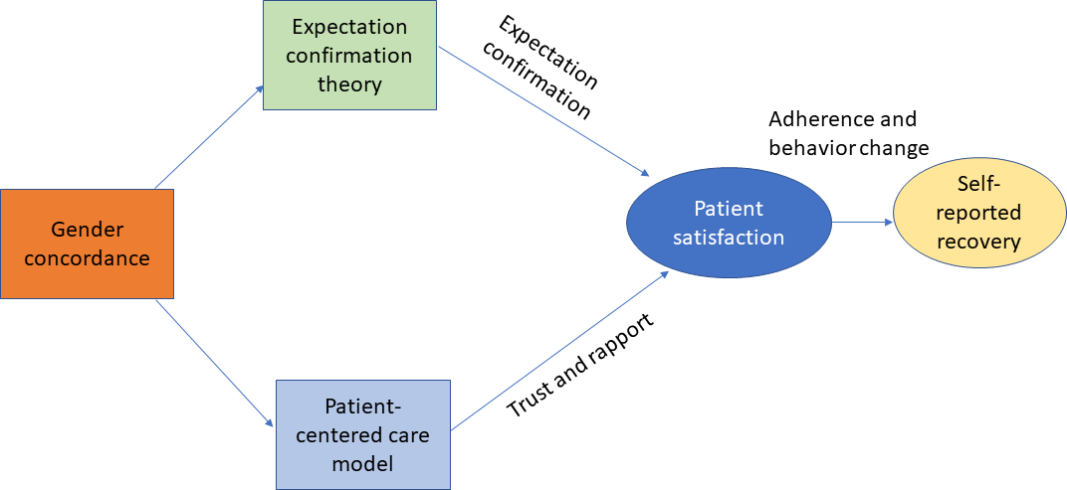
Theoretical framework.

PCCM emphasizes aligning care with patient preferences, values, and context [23,34]. In telemedicine, where nonverbal cues are limited, empathy, trust, and communication quality become more crucial than demographic similarity. This helps explain why patient satisfaction, rather than GC, was the strongest predictor of self-reported recovery [17]. Figure 1 illustrates how these 2 broad theories influence patient satisfaction and self-reported health outcomes through communication and trust.

### Comparison With Prior Work

These findings suggest telemedicine platforms, especially catering to Indian clients, should not default to GC-based matching. Instead, emphasis should be placed on enhancing communication skills and digital empathy, particularly in sensitive specialties such as gynecology. Male doctors are considered to be good at the technical skills and, hence, the trust and satisfaction [32,33]; and female doctors turn out to be more empathetic and better at communication, resulting in higher satisfaction [16,17]. Unlike evidence from the developed world, in the case of the Indian telemedicine context, GC is not playing any significant role. Hence, the training in active listening and patient engagement can be preferred tools over demographic alignment through matching algorithms [35].

Moreover, patient preferences, specialty, and combinations of them play an important role in final satisfaction and health outcome—recovery rate. The patient preferences should guide provider matching, particularly in specialties involving intimate care, without enforcing rigid defaults.

### Strengths and Limitations

This study is among the first to explore GC and patient-reported outcomes using a very large real-world dataset from India, covering multiple specialties. Its strength lies in its scale, cross-specialty comparisons, and integration of both satisfaction and recovery indicators.

However, several limitations must be noted. First, PRME data represented <1% of consultations, limiting statistical power. Second, pediatric cases may involve caregiver responses, introducing bias; hence, the study could not include it in the analysis. Third, recall and nonresponse bias could affect postconsultation surveys. Fourth, this was a single-platform dataset, limiting generalizability. Moreover, generalizability to a very different country would also be limited, as the current evidence suggests that the Indian context behaved differently compared to Western evidence. Fifth, gender identity was operationalized as binary sex due to data limitations. The current findings are presented keeping in mind all these limiting factors. So, there is a need for further research, as described previously.

### Future Directions

Further research is warranted to explore intersectional factors and evaluate objective health outcomes beyond self-reports. There is a definite need to also assess similar hypotheses of GC on health outcomes in physical outpatient department settings for understanding Indian evidence. Specialty-level research and insights would help inform not only patients but also the current medical students choosing their specialization and super-specialization. Future research should replicate this analysis across multiple telemedicine platforms, incorporating both quantitative and qualitative approaches to capture patients’ subjective experiences. Including gender identity, language preference, and digital literacy could offer richer insights into patient-provider rapport.

Longitudinal or experimental designs could further assess how targeted interventions, such as empathy training or communication workshops for physicians, impact satisfaction and clinical outcomes.

### Conclusions

This study examined how matching the gender of patients and doctors influenced satisfaction and patient-reported recovery rate, drawing on data from more than 286,000 telemedicine consultations across various medical specialties in India. The GC did not consistently predict recovery but did influence satisfaction. In fact, at the overall level, GC between patients and doctors resulted in lower satisfaction.

Patient satisfaction emerged as the strongest factor linked to self-reported recovery, underscoring how crucial trust, empathy, and clear communication are in virtual consultations [36]. However, because only a small fraction of consultations, less than 1% of the total data, included recovery data, conclusions drawn about health outcomes should be viewed with caution.

These findings suggest that Indian telemedicine platforms should prioritize strategies that enhance communication skills, cultural sensitivity, and patient education. Indian patients apparently showed that gender-discordant dyads would be more effective. In specialties such as gynecology, where GC showed more pronounced effects on health outcomes, customizable matching features or patient-preference options may be warranted.

The current study has implications for telemedicine platforms, especially those catering to the Indian population. The evidence suggests that, unlike the Western world, patient satisfaction and health outcomes are not influenced by the GC; hence, there is no support for matching algorithms to consider this as a variable. The doctors need to be aware of varying preferences as per patient gender. There is also a case of further research on specialty-level evidence that may inform future doctors to choose their specializations. This may lead to a more effective patient-centric health care delivery.

In summary, while GC is not a major factor across the board in Indian telemedicine, it does matter in specific contexts. Its impact depends on the medical specialty, cultural expectations, and the quality of communication, highlighting the importance of personalized approaches to patient-provider interactions.

## Abbreviations

CSAT: customer satisfaction
ECT: expectation confirmation theory
GC: gender concordance
OR: odds ratio
PCCM: patient-centered care model
PRME: patient-reported medical efficacy
